# Draft genome sequences of *Cylindrospermopsis raciborskii* strains CS-508 and MVCC14, isolated from freshwater bloom events in Australia and Uruguay

**DOI:** 10.1186/s40793-018-0323-1

**Published:** 2018-10-12

**Authors:** Juan J Fuentes-Valdés, Katia Soto-Liebe, Danilo Pérez-Pantoja, Javier Tamames, Lucy Belmar, Carlos Pedrós-Alió, Daniel Garrido, Mónica Vásquez

**Affiliations:** 1 Department of Molecular Genetics and Microbiology, Pontificia Universidad Católica de Chile, 8331150 Santiago, Chile; 2 Department of Chemical and Bioprocess Engineering, Pontificia Universidad Católica de Chile, 7820436 Santiago, Chile; 3 Programa Institucional de Fomento a la Investigación, Desarrollo e Innovación, Universidad Tecnológica Metropolitana, 8940577 Santiago, Chile; 4 Systems Biology Program, CNB, CSIC, Calle Darwin 3, 28049 Madrid, Spain

**Keywords:** Cylindrospermopsis, Bloom, Cyanobacteria, Environmental toxicity, Non-ribosomal peptide-synthetase, Polyketide synthases

## Abstract

Members of the genus *Cylindrospermopsis* represent an important environmental and health concern. Strains CS-508 and MVCC14 of *C. raciborskii* were isolated from freshwater reservoirs located in Australia and Uruguay, respectively. While CS-508 has been reported as non-toxic, MVCC14 is a saxitoxin (STX) producer. We annotated the draft genomes of these *C. raciborskii* strains using the assembly of reads obtained from Illumina MiSeq sequencing. The final assemblies resulted in genome sizes close to 3.6 Mbp for both strains and included 3202 ORFs for CS-508 (in 163 contigs) and 3560 ORFs for MVCC14 (in 99 contigs). Finally, both the average nucleotide identity (ANI) and the similarity of gene content indicate that these two genomes should be considered as strains of the *C. raciborskii* species.

**Electronic supplementary material:**

The online version of this article (10.1186/s40793-018-0323-1) contains supplementary material, which is available to authorized users.

## Introduction

Cyanobacterial bloom-forming species are a persistent global problem [1, 2]. *Cylindrospermopsis* *raciborskii*, is a species responsible for algal blooms that cause serious problems because of the wide variety of toxic compounds that it produces [3, 4]. Animal consumption of contaminated water with toxic metabolites produces symptoms associated with dermal rash, neural disturbance, hepatic and digestive disorder, and in some cases causing death [4, 5]. *C. raciborskii* was first described in Java (Indonesia) in 1912 [6], and was morphologically characterized in 1972 by Seenayya and Subba-Raju [7] as a Gram-negative-like, cylindrical filament able to fix nitrogen. To date, this species has been characterized as a producer of saxitoxin, a neurotoxin able to block voltage dependent mammalian sodium channels [8]. It also produces cylindrospermopsin, a toxin related with phosphatase metabolic inhibition in hepatocyte cells [9]. Recently, an anti-fungal glycolipopeptide affecting the plasma membrane integrity of *Candida albicans* cells, classified as hassallidin, has also been identified [10–12].

**Fig. 1:**
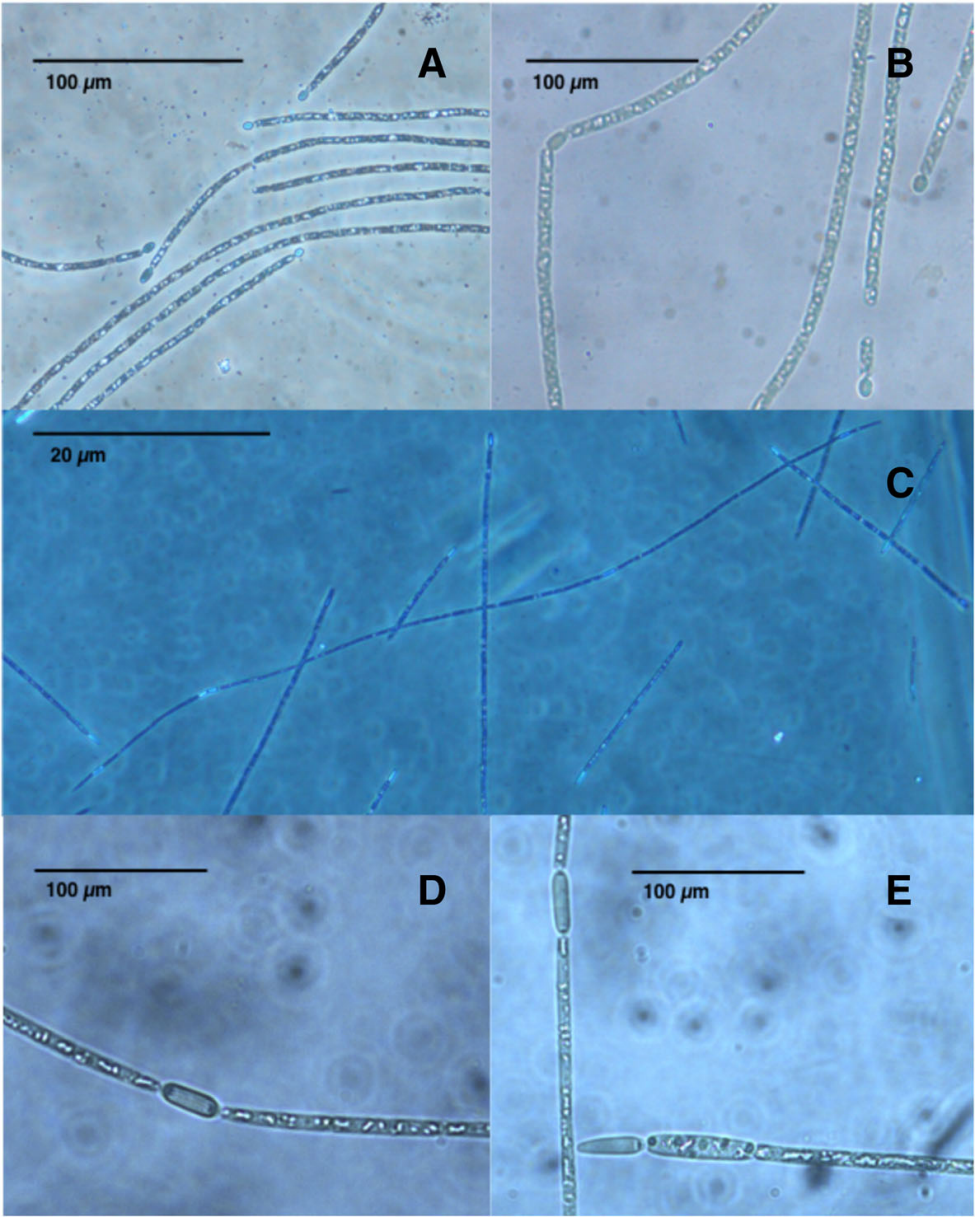
Light microscope photographs  of source organisms. **a**-**b** *C. raciborskii* CS-508 and of **c**-**e** *C. raciborskii* MVCC14

In order to understand the mechanisms responsible for the synthesis of these toxins, representative strains of this species have been characterized both genetically and chromatographically [13]. To date, Australian isolates have been characterized as CYL producers (CS-505 and CS-506), HAS producers (CS-505 and CS-509) and as non-toxin producers (CS-508) (unpublished data). In addition, the Uruguayan strain MVCC14 has been described as a STX producer [14]. Moreover, a Brazilian isolate *Raphidiopsis brookii* D9, a species phylogenetically closely related to *C. raciborskii* (Fig. 1), has also been reported as a STX producer [15–17]. The complete genome of *C. raciborskii* CS-505 and draft genomes of strains CS-506, CS-509 and *R. brookii* D9 are currently available [16, 18].

To provide further data to better understand the genomics and physiology of *C. raciborskii*, including its high capacity for dispersal, we performed a genome sequence analysis of Australian strain CS-508 and Uruguayan strain MVCC14, including gene annotation using the Clusters of Orthologous Group (COG) database [19]. Moreover, we also conducted a comparative genome analysis on five *C. raciborskii* strains: CS-505, CS-506, CS-508, CS-509 and MVCC14, in addition to *R. brookii* D9 to identify common genes.

## Organism information

### Classification and features

*C. raciborskii* is a relevant environmental species causing harmful blooms in freshwater environments, with certain strains synthesizing toxins.

*C. raciborskii* species (Tables 1 and 2), were initially described as microorganisms growing in the tropics, however, they have been reported in temperate freshwaters [20]. As previously described [21], the cells belonging to the genus *Cylindrospermopsis * could either be cylindrical filaments with terminal nitrogen fixation structures (heterocysts) (Fig. 1a-e) or resistant cells (akinetes). Both structures could be differentiated under nutrient-deficient culture media. In heterocyst-forming cyanobacteria, heterocysts are distributed in semi-regular intervals along the filament or only in the terminal position. The presence of intercalated heterocysts in *C. raciborskii* has been rarely observed, and has been thus described as a species with terminal heterocysts [22]. However, we observed intercalated heterocysts in strain MVCC14 under nitrogen starvation and under different nitrogen conditions (Fig. 1c-e)*.* The distribution of the heterocysts along the filament has been the subject of research by comparing genetic and physiological traits between *Cylindrospermopsis * and *Anabaena*, as models of differential patterns [23, 24]. *Anabaena* sp. PCC7120 differentiates heterocysts after every 8 to 12 vegetative cells under nitrogen deprivation [23, 24]. We were able to observe heterocysts more frequently in some filaments; regularity between heterocyst cells was approximately of 30 neighboring vegetative cells (SD ± 7, 4). This is the first report showing the transient presence of intercalary heterocyst in this *C. raciborskii* strain and further research should help to understand the genetic control that regulates this sporadic distribution of heterocysts in this *C. raciborskii* strain.

**Table 1:** Classification and general features of *C. raciborskii* strain CS-508 according to MIGS designation [45]

MIGS ID	Property	Term	Evidence code^a^
	Classification	Domain *Bacteria*	TAS [46]
		Phylum *Cyanobacteria*	TAS [47]
		Class *Cyanophyceae*	TAS [47]
		Order *Nostocales*	TAS [47]
		Family *Aphanizomenonacea*	TAS [47]
		Genus *Cylindrospermopsis*	TAS [6]
		Species *Cylindrospermopsis * *raciborskii* *Strains: CS-508*	TAS [48]
	Gram stain	Negative	TAS [7]
	Cell shape	Filaments	
	Motility	Temporary-motile (Hormogonia)	
	Sporulation	None	TAS [49]
	Temperature range	Mesophile	TAS [6]
	Optimum temperature	25 °C	TAS [50]
	pH range; Optimum	pH 7.50–9.21; pH 8.33	
	Carbon source	Autotroph	TAS [21]
MIGS-6	Habitat	Freshwater	TAS [51]
MIGS-6.3	Salinity	0.4% NaCl (maximum)	IDA
MIGS-22	Oxygen requirement	Aerobic	NAS
MIGS-15	Biotic relationship	free-living	NAS
MIGS-14	Pathogenicity	non-pathogen	TAS [52]
MIGS-4	Geographic location	Isolated Solomon Dam, Australia	NAS
MIGS-5	Sample collection	1999	NAS
MIGS-4.1	Latitude	−18.7241	IDA
MIGS-4.2	Longitude	146.5938	TAS [53]
MIGS-4.4	Altitude	Unknown	TAS [53]

^a^Evidence codes - IDA: Inferred from Direct Assay; *TAS* Traceable Author Statement (i.e., a direct report exists in the literature), *NAS* Non-traceable Author Statement (i.e., not directly observed for the living, isolated sample, but based on a generally accepted property for the species, or anecdotal evidence). These evidence codes are from the Gene Ontology project [54]

**Table 2:** Classification and general features of *C. raciborskii* strain MVCC14 according to MIGS designation [45]

MIGS ID	Property	Term	Evidence code^a^
	Classification	Domain *Bacteria*	TAS [46]
		Phylum *Cyanobacteria*	TAS [47]
		Class *Cyanophyceae*	TAS [47]
		Order *Nostocales*	TAS [47]
		Family *Aphanizomenonacea*	TAS [47]
		Genus *Cylindrospermopsis*	TAS [6]
		Species *Cylindrospermopsis * *raciborskii* *Strains: MVCC14*	TAS [55]
	Gram stain	Negative	
	Cell shape	Filaments	TAS [7]
	Motility	Non-motile	
	Sporulation	None	TAS [49]
	Temperature range	Mesophile	TAS [6]
	Optimum temperature	25 °C	TAS [50]
	pH range; Optimum	pH 7.50–9.21; pH 8.33	
	Carbon source	Autotroph	TAS [21]
MIGS-6	Habitat	Fresh water	TAS [51]
MIGS-6.3	Salinity	0.4% NaCl (maximum)	IDA
MIGS-22	Oxygen requirement	Aerobic	NAS
MIGS-15	Biotic relationship	free-living	NAS
MIGS-14	Pathogenicity	Saxitoxin (STX)	TAS [52]
MIGS-4	Geographic location	Isolated Laguna Blanca, Uruguay	NAS
MIGS-5	Sample collection	Unknown	NAS
MIGS-4.1	Latitude	−34.8984	TAS [14]
MIGS-4.2	Longitude	−54.8369	TAS [14]
MIGS-4.4	Altitude	Unknown	NAS

^a^Evidence codes - IDA: Inferred from Direct Assay; *TAS* Traceable Author Statement (i.e., a direct report exists in the literature), *NAS* Non-traceable Author Statement (i.e., not directly observed for the living, isolated sample, but based on a generally accepted property for the species, or anecdotal evidence). These evidence codes are from the Gene Ontology project [54]

**Fig. 2:**
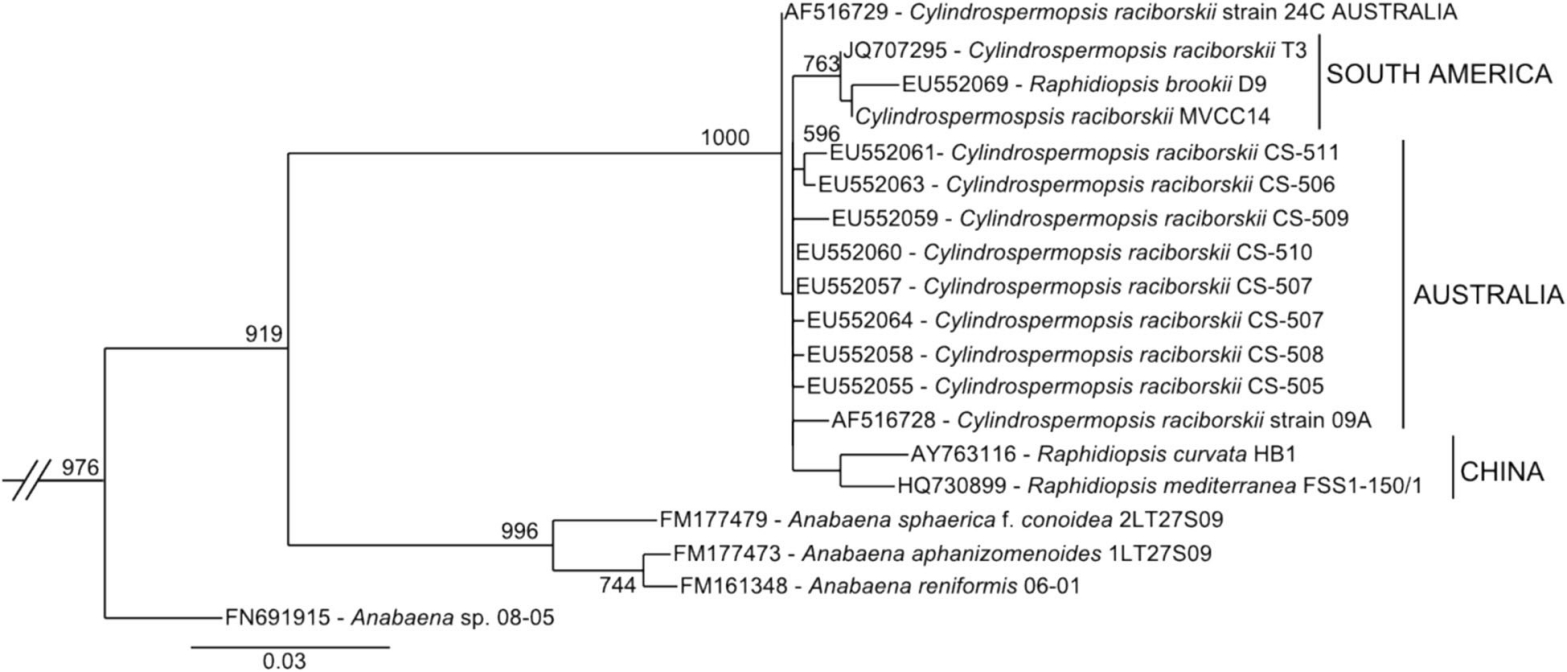
Maximum likelihood (ML) phylogenetic tree indicating the phylogenetic relationship of *C. raciborskii* strains. The ML tree is based on 16S rRNA gene sequences from *C. raciborskii* strains CS-508 and MVCC14 and sequences retrieved from previous reports stored in the NCBI database. These sequences were aligned using MUSCLE [43] and the phylogenetic tree was constructed with the phyML using GTR substitution model and BEST option for searching the starting tree [44]. Bootstrap support values ≥50% are indicated from 1000 bootstrap replicates. In supplemental material a complete phylogenetic tree is reported (Additional file 1: Figure S1)

Despite their very similar morphology, *C. raciborskii* and *R. brookii* have been classified as different species because the latter is unable of fix nitrogen and does not develop heterocysts (e.g. [25]). Here, the maximum likelihood phylogenetic tree of 16S-rRNA gene sequences shows that *R. brookii* and *C. raciborskii* strains constitute a statistically well-supported monophyletic clade (Fig. 2 and Additional file 1: Figure S1). This clade comprises sequences sharing ≥98% of similarity and show low evolutionary rate within the clade. Despite this, it is possible to identify some sub-clusters with a certain coherent phylo-geographical distribution as was previously described [26, 27]. For example, the sub-cluster comprising strains exclusively from South America (*R. brookii* D9, *C. raciborskii* MVCC14 and T3) is segregated with a well-supported statistical value (Fig. 2, Additional file 1: Figures. S2 and S4). Phylogenetic analyses from other phylogenetic markers also displayed the monophyletic nature among *R. brookii* and *C. raciborskii* strains (Additional file 1: Figures. S2, S3, S4 and S5). This is congruent with a previous study of phylogenetic relationships inferred from several conserved genes, which postulate that *Cylindrospermopsis * and *Raphidiopsis* representatives should be congeners [28]. However, to assess the taxonomic classification of these microorganisms further phylogenetic analyses (e.g., global genome comparisons) or more complete physiological descriptions are required.

## Genome sequencing information

### Genome project history

Strains CS-508 and MVCC14 were selected for sequencing based on their phylogenetic relationship between strains from South America and Australia. Sequenced draft genomes were annotated using RAST [29] The CS-508 Whole Genome Shotgun project has been deposited at DDBJ/ENA/GenBank under the accession MBQX00000000. The version described here is MBQX01000000. MVCC14 Whole Genome Shotgun Project has been deposited under the accession ID MBQY00000000. The version described in this paper is version MBQY01000000. A summary of the project information is shown in Table 3.

**Table 3:** Project information

MIGS ID	Property	Term (for CS-508)	Term (for MVCC14)
MIGS 31	Finishing quality	High- Quality Draft	High- Quality Draft
MIGS-28	Libraries used	Illumina	Illumina
MIGS 29	Sequencing platforms	Illumina HiSeq2000	Illumina HiSeq2000
MIGS 31.2	Fold coverage	20×	20×
MIGS 30	Assemblers	IDBA, SPADES, VELVET and ABYSS	IDBA, SPADES, VELVET and ABYSS
MIGS 32	Gene calling method	Rast	Rast
	Locus Tag	CYL_CS508	CYL_MVCC14
	GenBank ID	MBQX00000000	MBQY00000000
	GenBank Date of Release	November 01, 2016	November 01, 2016
	GOLD ID	Gs0120410	Gs0121371
	BIOPROJECT	PRJNA327084	PRJNA327088
MIGS 13	Source Material Identifier	Freshwater	Freshwater
	Project relevance	Environment	Environment

### Growth conditions and genomic DNA preparation

*C. raciborskii* cultures were grown in MLA medium [30], under 12:12 light:dark cycles at 25 °C. Total DNA extractions were carried out using 100 mL of exponential growth culture, obtaining approximately 1 g of wet cell pellet. DNA purification was conducted by standard CTAB protocol [31]. Total cell pellets were mechanically disrupted and resuspended in 500 μL of CTAB buffer, and incubated at 55 °C for 1 h under constant mixing. The DNA was purified using 500 μL phenol/chloroform/isoamyl alcohol (25:24:1) and centrifuged at 8000 x g for 7 min. DNA was precipitated using isopropanol/ammonium acetate (0.54 vol cold isopropanol, 0.08 vol ammonium acetate 7.5 M). Finally, DNA was washed with 70% and then with 90% ethanol and resuspended in 50 μL of pure water. DNA extraction was visualized using red gel staining in a 1% agarose gel under UV light.

### Genome sequencing and assembly

Both genomes were obtained by a shotgun strategy using Illumina MiSeq sequencing technology. A total of 8,308,910 paired-end reads were obtained for CS-508 strain and 28,711,437 paired-end reads for MVCC14 strain. Quality control checks were performed on the raw FASTQ data using FastQC (version 0.10.1) [32]. Sequencing reads were trimmed for sequencing adaptors using Trimmomatic (version 0.32) [33] and the quality filtering and trimming was done by Prinseq-lite (version 0.20.4) [34]. Briefly, reads were trimmed for ‘N’ characters and low quality nucleotides (Phred score cutoff of 24) and then any read with an average Phred score below 29 and shorter than 80 nt was discarded. A de novo assembly strategy involving multiple algorithms and merging of the individual assemblies was performed. Assemblies by IDBA [35], SPADes [36], VELVET [37] and ABYSS [38] algorithms were generated by using the platform MIX software [39] to improve draft assembly by reducing contig fragmentation. Contigs shorter than 1000 bp were discarded. The final assembly resulted in 163 contigs for CS-508 and 99 contigs for MVCC14, accounting for 3,558,956 bp and 3,594,524 bp, respectively. CheckM analysis [40] indicated a genome completeness of 97.57% for CS-508 and 96.29% for MVCC14.

### Genome annotation

The gene annotation process was conducted using the RAST Server 2.0 [29]. Predicted coding sequences were extracted from RAST platform and homology was evaluated by BLASTp scan, with each predicted ORF as a query against the complete bacterial database.

## Genome properties

*C. raciborskii* CS-508 and MVCC14 draft genomes have a GC% content of 43 and 44 respectively (Table 4), containing 3202 and 3560 ORFs each. Table 5 shows the COG distribution of the corresponding genes. A high number of these encode metabolic proteins (COG codes R, S, M, C, E, P, O, H and T). Interestingly, no genes for the “RNA processing and modification” category were found in any genome. This has been observed in another cyanobacterial genome [41] and could be explained by genetic divergence of these cyanobacteria. Approximately 22% (CS-508) and 26% (MVCC14) of the total coding genes were not classified in any COG category.

**Table 4:** Genome statistics of CS-508 (A) and MVCC14 (B)

Attribute	A Value	A % of Total	B Value	B % of Total
Genome size (bp)	3,558,956	100	3,594,524	100
DNA coding (bp)	3,039,246	85.34	3,074,946	85.55
DNA G + C (bp)	1,530,351	43	1,581,591	44
DNA scaffolds	163	100	99	100
Total genes	3344	100	3616	100
Protein coding genes	3302	98.74	3560	98.45
RNA genes	42	1.26	56	1.55
Pseudo genes	–	–	–	–
Genes in internal clusters	–	–	–	–
Genes with function prediction	2247	67.19	2337	64.63
Genes assigned to COGs	1747	56.16	1796	55.55
Genes with Pfam domains	2656	79.43	2800	77.43
Genes with signal peptides	71	2.12	63	1.74
Genes with transmembrane helices	255	7.63	748	20.66
CRISPR repeats	7	–	9	–

**Table 5:** Number of genes associated with general COG functional categories

	CS-508		MVCC14		Description
Code	Value	%age	Value	%age	
J	142	4.56	143	4.37	Translation, ribosomal structure and biogenesis
A	0	0.00	0	0.00	RNA processing and modification
K	69	2.22	64	1.96	Transcription
L	88	2.83	112	3.43	Replication, recombination and repair
B	0	0.00	0	0.00	Chromatin structure and dynamics
D	21	0.68	19	0.58	Cell cycle control, Cell division, chromosome partitioning
V	0	0.00	0	0.00	Defense mechanisms
T	49	1.58	54	1.65	Signal transduction mechanisms
M	123	3.95	130	3.98	Cell wall/membrane biogenesis
N	6	0.19	5	0.15	Cell motility
U	0	0.00	0	0.00	Intracellular trafficking and secretion
O	111	3.57	111	3.40	Posttranslational modification, protein turnover, chaperones
C	157	5.05	163	4.99	Energy production and conversion
G	99	3.18	93	2.84	Carbohydrate transport and metabolism
E	125	4.02	123	3.76	Amino acid transport and metabolism
F	45	1.45	44	1.35	Nucleotide transport and metabolism
H	104	3.34	107	3.27	Coenzyme transport and metabolism
I	32	1.03	31	0.95	Lipid transport and metabolism
P	128	4.11	130	3.98	Inorganic ion transport and metabolism
Q	40	1.29	36	1.10	Secondary metabolites biosynthesis, transport and catabolism
R	252	8.10	262	8.01	General function prediction only
S	156	5.01	169	5.17	Function unknown
–	1364	43.84	1473	45.06	Not in COGs

The total is based on the total number of protein coding genes in the genome

## Insights from the genome sequence

Photoautotrophic metabolic pathways were reconstructed in CS-508 and MVCC14 draft genomes, based on the predicted metabolic pathways in previous sequenced genomes of *C. raciborskii* [16, 18]. Nitrogen metabolic systems related to ammonium, nitrate and nitrite acquisition genes, as well as heterocyst differentiation and nitrogen fixation, were identified in both genome drafts.

Sequenced genomes were compared to previously published *C. raciborskii* and *R. brookii* genomes. We determined the average nucleotide identity in these genomes by a two-way comparison analysis (Table 6), using the inference tool ANI calculator [20]. The percentage of shared genes between strains ranged from 93.23 to 99.77%. According to the ANI value, the complete group, *C. raciborskii* and *R. brookii* could be considered as members of the same species, considering a threshold value of 95% [42].

**Table 6:** Average nucleotide identity (ANI) values for the sequenced *C. raciborskii* and *Raphidiopsis brookii* strains

	*Cylindrospermopsis * CS-505	*Cylindrospermopsis * CS-506	*Cylindrospermopsis * CS-508	*Cylindrospermopsis * CS-509	*Cylindrospermopsis * MVCC14	*R. brookii* D9
*Cylindrospermopsis * CS-505	–	99.31	99.73	99.77	93.73	93.26
*Cylindrospermopsis * CS-506	99.31	–	99.39	99.32	93.45	92.85
*Cylindrospermopsis * CS-508	99.73	99.39	–	99.76	93.82	93.25
*Cylindrospermopsis * CS-509	99.77	99.32	99.76	–	93.80	93.23
*Cylindrospermopsis * MVCC14	93.73	93.45	93.82	93.80	–	97.17
*R. brookii* D9	93.26	92.85	93.25	93.23	97.17	–

We identified four genes encoding a non-ribosomal peptide synthetase complex in the CS-508 genome related to the hassallidin biosynthesis. We found in CS-508 the same gene cluster as in the hassallidin producers CS-509, CS-505 and *Anabaena* SYKE748A [10, 16, 18], with no evidence of mutations in the hassallidin cluster. Surprisingly, we were not able to detect the presence of hassallidin in CS-508 cultures, according to LC-MS/MS analysis (unpublished results). In the MVCC14 draft genome, we identified a group of genes related to STX biosynthesis. STX is a paralytic biotoxin produced by marine dinoflagellates and freshwater cyanobacteria [14]. The *sxt* gene cluster found in MVCC14 has a similar distribution and toxin profile to *R. brookii* D9 [16]. We did not find NRPS sequences in the MVCC14 genome.

## Conclusions

In order to understand the genomics of the toxin producing, bloom forming *C. raciborskii**,* this work presents two drafts of sequenced genomes from the non-toxic Australian strain CS-508 and the Uruguayan neurotoxin-producer strain MVCC14. An NRPS gene cluster related with hassallidin production was identified in CS-508 and PKS-like set of genes related with STX production was identified in the genome of the MVCC14 strain. Considering the 16S rRNA gene phylogenetic analysis and genome level comparison, we identified a phylogeographical segregation of the *C. raciborskii* and *R. brokii* strains retrieved from South America. Disregarding nitrogen fixation ability, these results suggest *R. brookii* D9 and *C. raciborskii*mvcc14 are closely related at genome level, which could lead to new research to corroborate the *Cylindrospermopsis **/Raphidiopsis* clade as one comprised by two genera or by a single genus with different species.

## Additional file

Additional file 1: **Figure S1.** Cyanobacterial ML phylogenetic tree based on 16S rRNA gene sequences. **Figure S2.** ML phylogenetic tree based on *rbcL* gene sequences from relatives cyanobacteria. F**igure S3.** ML phylogenetic tree based on ribulose-1,5-bisphosphate carboxylase/oxygenase large subunit (RbcL) proteins from relatives cyanobacteria. **Figure S4.** ML phylogenetic tree based on *psbA* gene sequences from relatives cyanobacteria. **Figure S5.** ML phylogenetic tree based on Photosystem II D1 (PsbA) proteins from relatives cyanobacteria. (DOCX 979 kb)
