# Supplementary material for: Draft genome sequences of Cylindrospermopsis raciborskii strains CS-508 and MVCC14, isolated from freshwater bloom events in Australia and Uruguay
Source: Stand Genomic Sci. 2018 Oct 12;13:26. doi: 10.1186/s40793-018-0323-1 (PMC6186047; doi:10.1186/s40793-018-0323-1)
Supplement: Supplementary file 1 — Figure S1. Cyanobacterial ML phylogenetic tree based on 16S rRNA gene sequences. Figure S2. ML phylogenetic tree based on rbcL gene sequences from relatives cyanobacteria. Figure S3. ML phylogenetic tree based on ribulose-1,5-bisphosphate carboxylase/oxygenase large subunit (RbcL) proteins from relatives cyanobacteria. Figure S4. ML phylogenetic tree based on psbA gene sequences from relatives cyanobacteria. Figure S5. ML phylogenetic tree based on Photosystem II D1 (PsbA) proteins from relatives cyanobacteria. (DOCX 979 kb) [file 40793_2018_323_MOESM1_ESM.docx]

**Supplemental figure 1**. Cyanobacterial ML phylogenetic tree based on 16S rRNA gene sequences. The alignment was achieved by MUSCLE [1] and filtered by Gblocks [2,3]. The phylogenetic tree of the unambiguously aligned sequences (1287 bp) was constructed with phyML algorithm using GTR substitution model and BEST option [4]. Bootstrap support values ≥50% are indicated from 1000 replicates. (Note: the box highlights the branch of this tree reported in the main article)


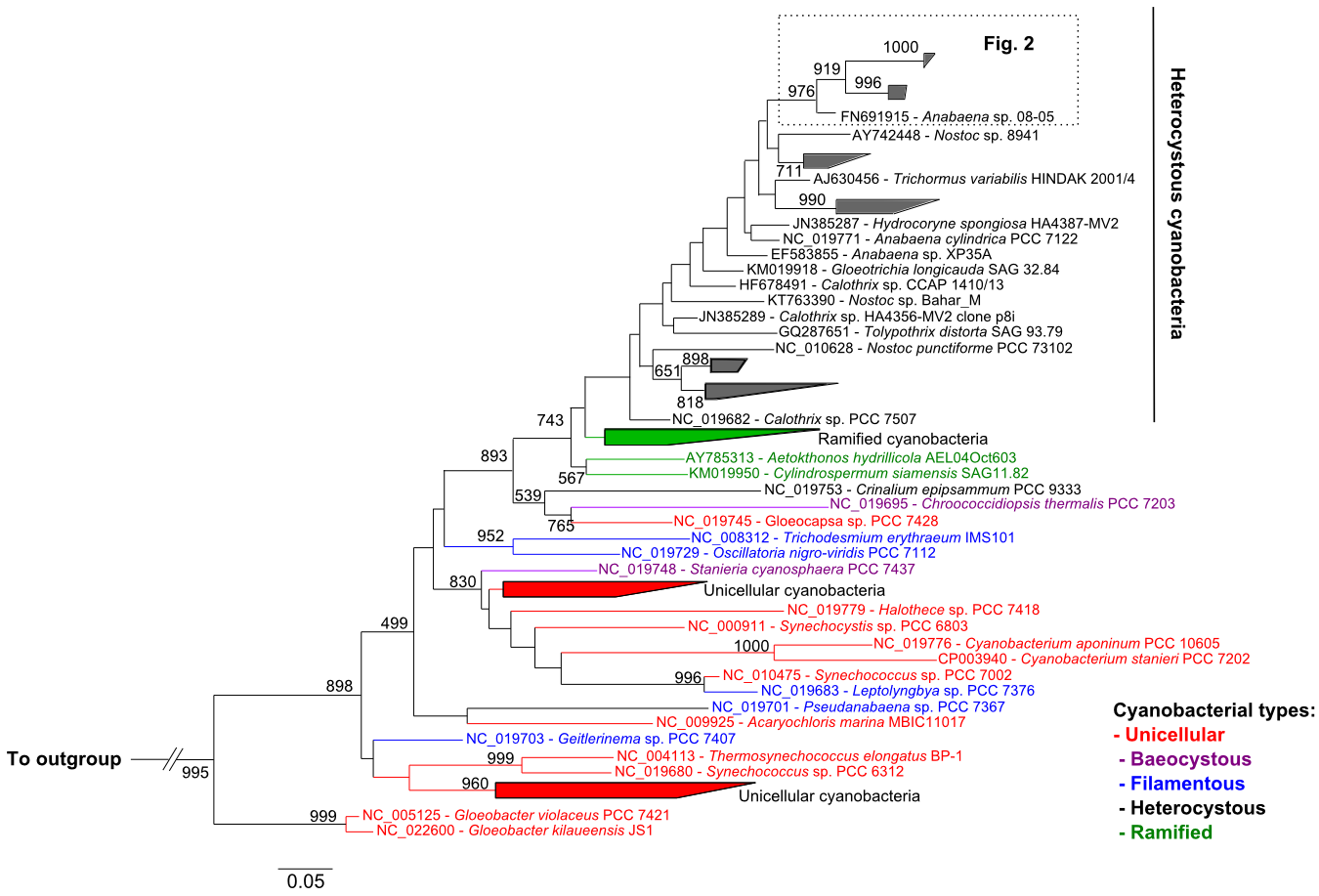


**Supplemental figure 2**. ML phylogenetic tree based on *rbcL* gene sequences from relatives cyanobacteria. The alignment was achieved by MUSCLE [1] and filtered by Gblocks [2,3]. The phylogenetic tree of unambiguously aligned sequences (1387 bp) was constructed with the phyML algorithm using GTR substitution model and BEST option [4]. Bootstrap support values ≥50% are indicated from 1000 replicates.


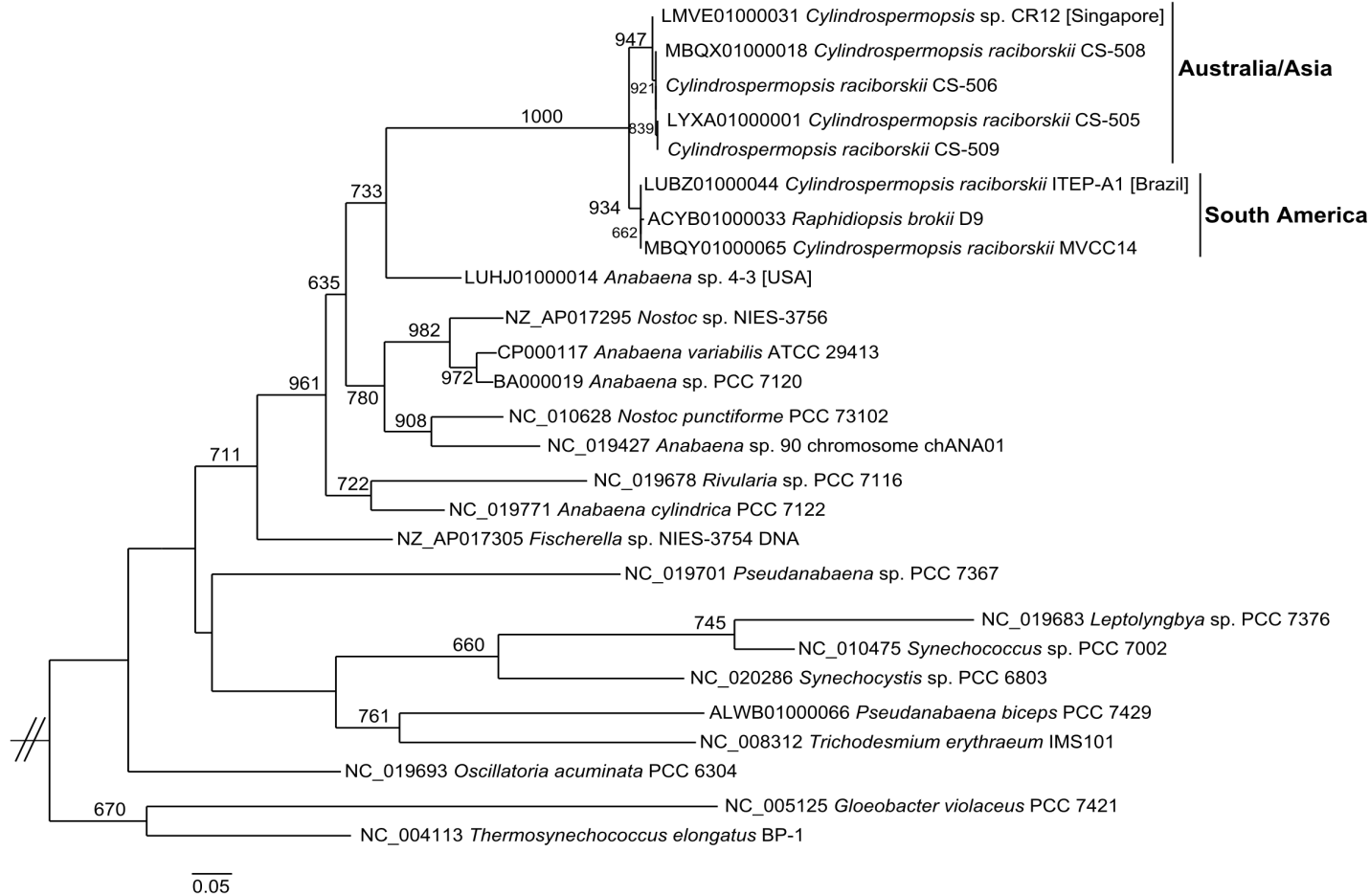


**Supplemental figure 3**. ML phylogenetic tree based on ribulose-1,5-bisphosphate carboxylase/oxygenase large subunit (RbcL) proteins from relatives cyanobacteria. The alignment was achieved by MUSCLE [1] and filtered by Gblocks [2,3]. The phylogenetic tree of unambiguously aligned sequences (462 Aa) was constructed with the phyML algorithm using LG substitution model and BEST option [4]. Bootstrap support values ≥50% are indicated from 1000 replicates.


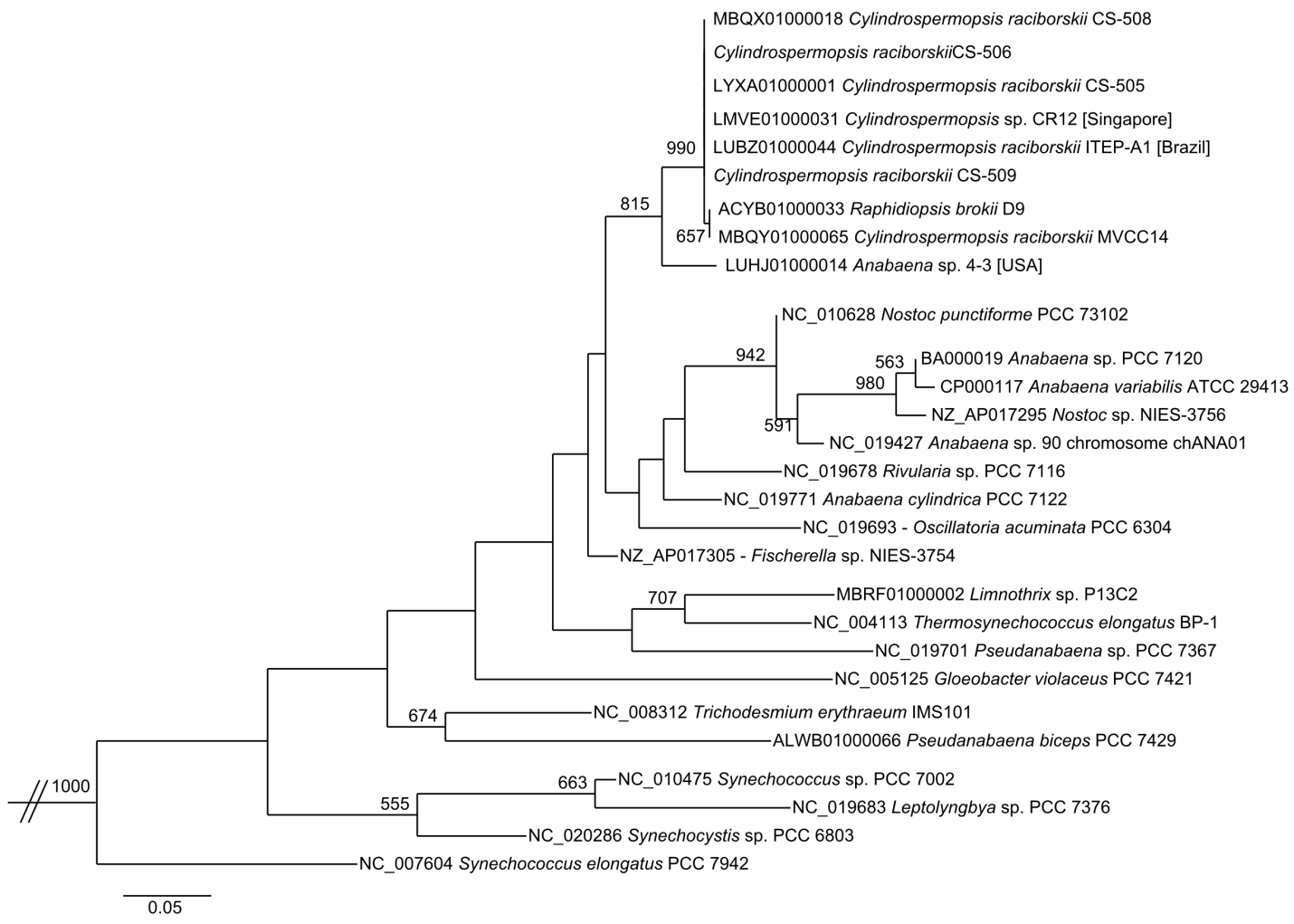


**Supplemental figure 4**. ML phylogenetic tree based on *psbA* gene sequences from relatives cyanobacteria. The alignment was achieved by MUSCLE [1] and filtered by Gblocks [2,3]. The phylogenetic tree of unambiguously aligned sequences (875 bp) was constructed with the phyML algorithm using GTR substitution model and BEST option [4]. Bootstrap support values ≥50% are indicated from 1000 replicates.

**
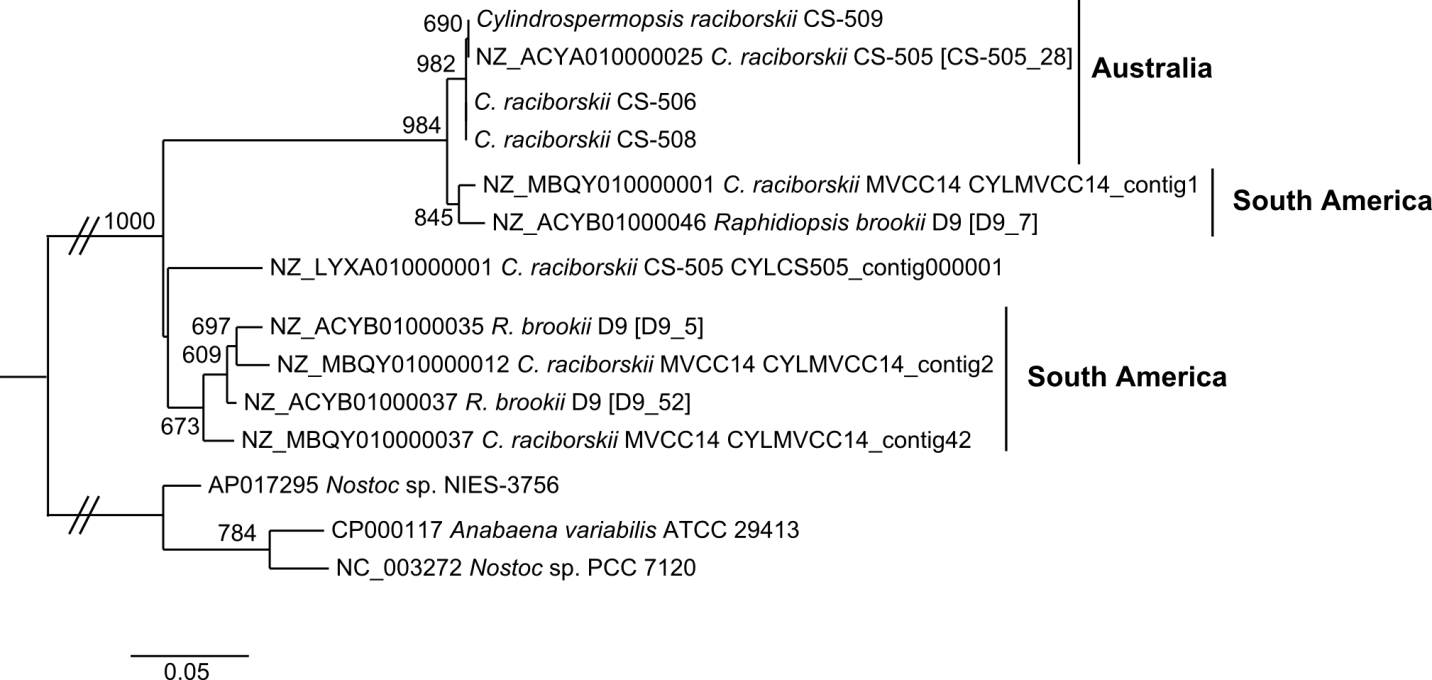
**

**Supplemental figure 5**. ML phylogenetic tree based on Photosystem II D1 (PsbA) proteins from relatives cyanobacteria. The amino acid sequences were aligned by MUSCLE [1]. This alignment does not show poorly conserved regions. The phylogenetic tree of this unambiguously aligned sequences (361 Aa) was constructed with the phyML algorithm using LG substitution model and BEST option [4]. Bootstrap support values ≥50% are indicated from 1000 replicates.


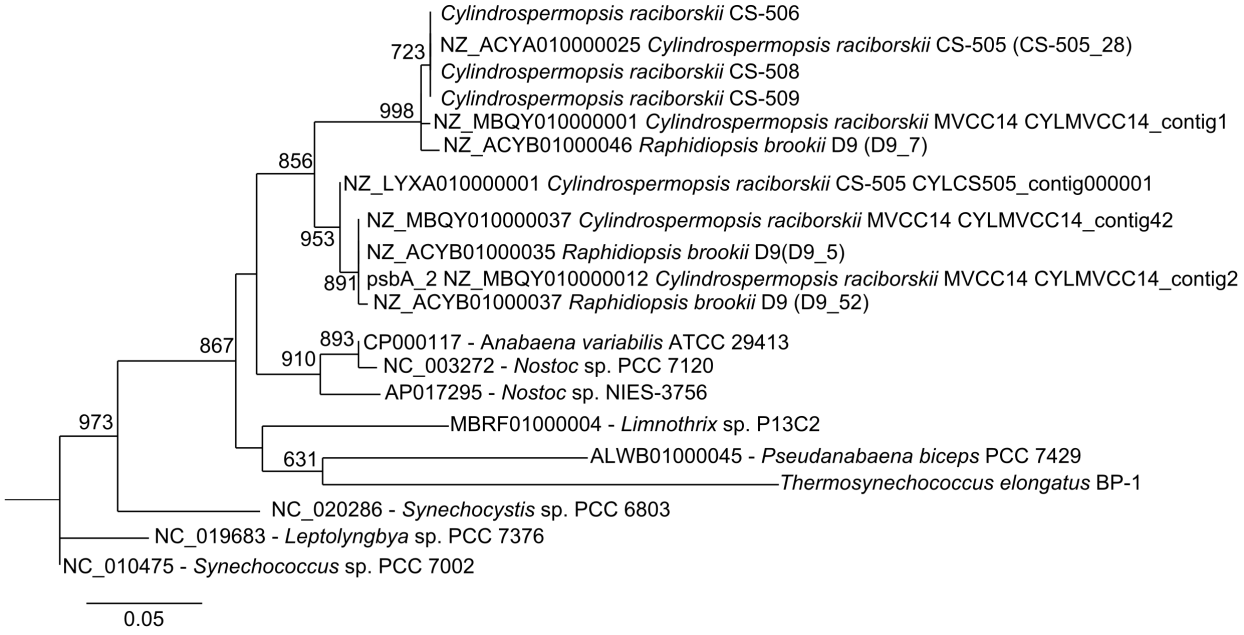


**References**

1. Edgar RC. MUSCLE: Multiple sequence alignment with high accuracy and high throughput. Nucleic Acids Res. 2004;32:1792–7.

2. Castresana J. Selection of Conserved Blocks from Multiple Alignments for Their Use in Phylogenetic Analysis. Mol. Biol. Evol. 2000;17:540–52.

3. Talavera G, Castresana J. Improvement of phylogenies after removing divergent and ambiguously aligned blocks from protein sequence alignments. Syst. Biol. 2007;56:564–77.

4. Guindon S, Dufayard J-F, Lefort V, Anisimova M, Hordijk W, Gascuel O. New algorithms and methods to estimate maximum-likelihood phylogenies: assessing the performance of PhyML 3.0. Syst. Biol. 2010;59:307–21.
